# Robot-Assisted Excision of a Pararectal Gastrointestinal Stromal Tumor in a Patient with Previous Ileal Neobladder

**DOI:** 10.1155/2014/632852

**Published:** 2014-09-01

**Authors:** A. Ploumidis, A. Mottrie, A. F. Spinoit, M. Gan, V. Ficarra, R. Andrianne

**Affiliations:** ^1^Department of Urology, OLV Vattikuti Robotic Surgery Institute, Moorselbaan 164, 9300 Aalst, Belgium; ^2^Department of Urology, Ghent University Hospital, De Pintelaan 185, 9000 Ghent, Belgium; ^3^Department of Urology, University of Padova, Via Giustiniani, 2, 35100 Padova, Italy; ^4^Department of Urology, CHU Liege, Domaine Universitaire du Sart Tilman, 4000 Liège, Belgium

## Abstract

Gastrointestinal stromal tumors (GISTs) are the most frequent mesenchymal tumors of the gastrointestinal tract with surgical resection remaining the cornerstone of therapy. Pararectal lesions are considered to be technically difficult and pose in some cases a challenge. We report, to the best of our knowledge, the first robotic-assisted pararectal GIST excision. A 43-year-old man was referred to our center with pararectal GIST recurrence, despite treatment with targeted therapy. Eleven years ago, he underwent extensive abdominal surgery including cystoprostatectomy with ileal neobladder diversion due to GIST resection in the rectoprostatic space. Robot-assisted surgical resection was successfully performed without the need for temporary colostomy. The postoperative course of the patient was uneventful, and the pathology report confirmed a GIST recurrence with negative surgical margins and pelvic lymph nodes free of any tumor. Robotic-assisted pelvic surgery can be extended to incorporate excision of pararectal GISTs, as a safe, less invasive surgical alternative with promising oncological results and minimal injury to adjacent structures.

## 1. Introduction 

Gastrointestinal stromal tumors (GISTs) are the most frequent mesenchymal tumors of the gastrointestinal tract with an incidence of 3300–4350 cases per year in the United States. The most common sites of occurrence are the stomach (60%) and the small intestine (30%), while about 5% originate in the colon and rectum. Growing knowledge of the pathogenesis of the disease and targeted molecular therapies have revolutionized the treatment of rectal GISTs, though surgical excision still remains the mainstay of therapy [1, 2].

Rectal lesions are considered to be technically difficult and pose in some cases a challenge to the surgeon due to the confined space of the pelvis combined with the inherent capability of the tumor to adhere to adjacent structures or even the pelvic floor [3, 4]. However, advantages of the robotic platform, such as the magnified visual field accompanied by the wristed instrumentation, can facilitate radical resection with minim tissue trauma. We report to the best of our knowledge, the first robotic-assisted pararectal GIST excision in a patient with previous extensive abdominal surgery.

## 2. Case Report

A 43-year-old man was referred to our institution with a pararectal recurrence of a GIST. In 2001, he underwent an initial resection of a GIST localized in the rectoprostatic space. This procedure resulted in an open partial rectal resection with temporary colostomy and a cystoprostatectomy with creation of an ileal neobladder. Since 2009, he was followed for a pararectal lesion suspicious for recurrence, which was confirmed with biopsies. Despite the initial treatment with imatinib mesylate, progressive tumor growth occurred. The patient placed a high priority on his quality of life and was reluctant to any open procedure that would likely end up in a temporary diverting colostomy. Therefore, the patient was planned for robot-assisted tumor enucleation in an attempt to minimize collateral damage.

**Figure 1:**
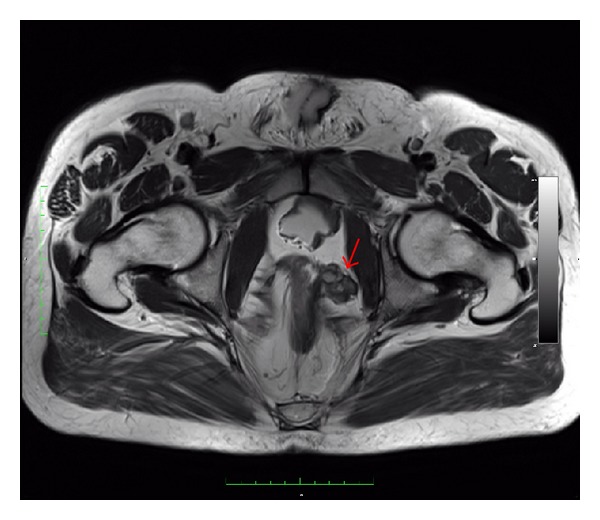
Pelvic MRI of the patient. The arrow demonstrates the pararectal GIST.

**Figure 2:**
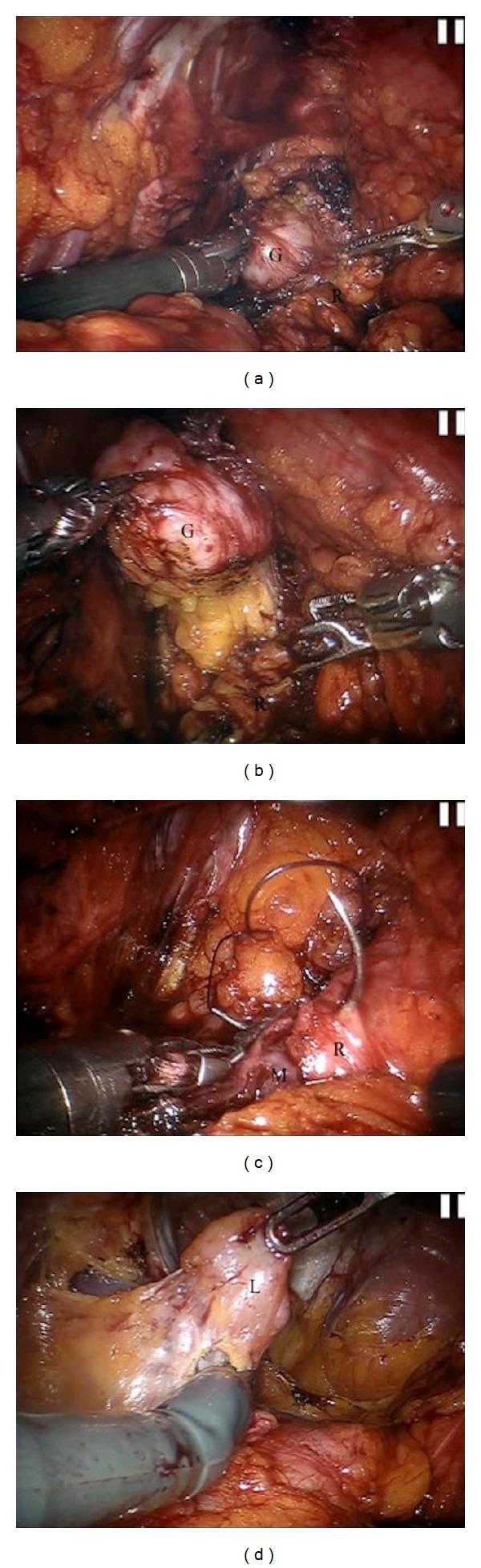
(a) The tumor is identified at the left lateral wall of the rectum. (b) The GIST is dissected free under direct vision with safe margins. (c) Suturing the rectal wall in a two-layer fashion with the mucosa of the rectum intact. (d) Resection of suspicious pelvic lymph nodes. G: GIST, R: rectum, M: mucosa of the rectum, and L: pelvis lymph node.

The patient was bowel prepped the day before surgery. After endotracheal intubation, the patient was adequately padded, draped, and placed in a 30-degree trendelenburg position with the legs apart. Broad-spectrum antibiotics were administered and a bladder catheter was placed. Due to the previous cystoprostatectomy procedure with ileal neobladder diversion, adhesions were expected; thus, the camera port was placed with open technique and a thorough inspection of the abdomen was performed. Three robotic and two assisting trocars were inserted under vision, in a configuration similar to that used in robotic-assisted radical prostatectomy [5]. Anatomical orientation in an abdomen with previous extensive surgery was of paramount importance with the 3D robotic vision having evident advantages. Thorough adhesiolysis was performed and retrograde filling of the neobladder with normal saline aided in identifying its borders. Meticulous dissection was applied along the sigmoid, detaching it from the left lateral abdominal wall in an effort to keep it as a landmark and enter the true pelvis in a safe plane of dissection. MRI imaging through TILEPro (Intuitive Surgical, Inc.) technology was used to confirm the level of the tumor in the pelvis throughout the whole procedure (Figure 1). The fourth robotic instrument was used for traction, while countertraction was applied from the assistant with laparoscopic instruments. The tumor was located in the left lateral wall of the rectum and was dissected free under direct vision with safe margins from THE surrounding structures and the mucosa of the rectum avoiding any iatrogenic penetration of the pseudocapsule (Figures 2(a) and 2(b)). At the same time, care was taken not to further injure the rectal wall and enter the rectal lumen. Retrograde insufflation of the rectum with air, while filling the pelvis with water, confirmed no rectal leak. Consequently, the rectal defect was sutured with 3-0 VICRYL (Ethicon Inc.,) in a two-layer fashion (Figure 2(c)). Suspiciously, enlarged lymph nodes that were noted along the left obturator fossa and hypogastric artery were resected for pathology (Figure 2(d)). No temporary diverting colostomy was offered. After confirming hemostasis, a drain was placed in the pelvis and a laparoscopic entrapment bag was used to retrieve the specimen.

Console time was 130 min and estimated blood loss was 200 cc. No intraoperative complications were encountered. Convalescence was uneventful with the patient passing flatus on day one. Clear liquids were consumed on the first postoperative day, which was switched to a fiber-free diet for 7 days before starting a normal diet. The patient was discharged the third day postoperatively. The histopathology report revealed a GIST, with a maximum diameter of 3.3 cm consisting of a proliferative cell-rich component with spindle cells and areas of hemorrhage and necrosis. The other component was cell poor with mucoid degeneration and some vital tumor cells. The tumor cells were CD117- and CD34-positive. The surgical margins were negative and the lymph nodes were free of tumor as well.

## 3. Discussion

GISTs are mesenchymal tumors that arise from the intestinal cells of Cajal. These cells are part of the myenteric plexus of the gut wall and regulate the autonomic nerve system. They function as a pacemaker controlling the intestinal motility. On their cell surface, they express CD117 or c-KIT, which is a transmembrane receptor protein with tyrosine kinase function that regulates proliferation, differentiation, cell adhesion, and apoptosis. GISTs occur due to mutation on the c-KIT protein that leads to gain of function resulting in continuous activation of the protein and subsequent unregulated proliferation. Imatinib mesylate, a tyrosine kinase inhibitor, binds to the specific domain of the receptor causing regression of the CD117 positive GISTs [1, 2, 6].

The National Institute of Health Workshop has subdivided the GISTs based on the risk of malignant behavior. A cut-off point of 5 mitoses per 50 HPF is regarded as the upper limit for benign behavior, especially in gastric GISTs [7]. As far as the anorectal GISTs are concerned, this has been addressed in a study by Miettinen et al., where 144 cases of anorectal GISTs were prospectively analyzed. Tumors in the very low risk group (<2 cm of size and <5 mitoses/HPF) with low rate of recurrence and indolent behavior were considered suitable for local resection, while tumors with malignant behavior (>5 cm of size or >5 mitoses/50 HPF) had high rates of recurrence and metastasis [8].

Although, targeted therapy has given new perspective in the treatment of the disease, especially in the neoadjuvant and adjuvant setting, surgery remains the cornerstone of therapy for patients with primary GIST when being technically feasible with limited morbidity. Negative surgical margins and preventing intraoperative rupture of the tumor are of utmost importance, while lymph node dissection is not necessary, except in patients where suspicious nodes are encountered such as in our case. With regard to anorectal GISTs, local excision may be sufficient with a minimum risk of morbidity and preservation of the anal sphincter, while in some cases more extensive surgery is unavoidable [1, 6].

Small series of laparoscopic resection of gastric GISTs have been reported in the literature with good oncological results [9]. On the other hand, data on laparoscopic resection of rectal GISTs is sparse with few case reports published [3, 4, 10]. A common remark in these reports is the substantial advantage of the magnified visual field, which can potentially benefit the patient by less extensive resections. Recently, robot-assisted surgery has been implemented in the past for the resection of gastric GISTs. Most authors conclude that the wristed instrumentation combined with the enhanced vision aids in performing an oncologically safe resection while preserving adjacent tissue, even in large tumors or tumors with a difficult location [11–13]. In this case, a robotic approach was decided not only for the potential advantages of a less invasive procedure, but also for the induction of minimal collateral injury during access to the deep pelvis having always in mind the safety of the neobladder and its vasculature. The freedom of motion of the robotic arms is appreciated especially during suturing in difficult angles where ensuring reapproximation of the defect is important, thus avoiding a temporary colostomy.

## 4. Conclusion

Robotic surgery with the wristed instrumentation and three-dimensional vision has proven advantages compared to conventional laparoscopy, especially in confined spaces such as the pelvis. We report to the best of our knowledge the first robotic-assisted pararectal GIST excision. As demonstrated in this case, the indications for robotic-assisted pelvic surgery can be extended to incorporate excision of rectal GISTs, as a safe and minimal invasive surgical alternative with promising oncological results. In experienced hands, previous extended abdominal operations should not be considered an absolute contraindication for robotic surgery.
